# John Dee and the alchemists: Practising and promoting English alchemy in the Holy Roman Empire

**DOI:** 10.1016/j.shpsa.2011.12.009

**Published:** 2012-09

**Authors:** Jennifer M. Rampling

**Affiliations:** University of Cambridge, Department of History and Philosophy of Science, Free School Lane, Cambridge CB2 3RH, UK

**Keywords:** John Dee, Edward Kelley, George Ripley, Alchemy, Rudolf II, Prague, Manuscript circulation

## Abstract

This paper investigates John Dee’s relationship with two kinds of alchemist: the authorities whose works he read, and the contemporary practitioners with whom he exchanged texts and ideas. Both strands coincide in the reception of works attributed to the famous English alchemist, George Ripley (d. c. 1490). Dee’s keen interest in Ripley appears from the number of transcriptions he made of ‘Ripleian’ writings, including the *Bosome book*, a manuscript discovered in 1574 and believed to have been written in Ripley’s own hand. In 1583, Dee and his associate Edward Kelley left England for East Central Europe, taking with them a proportion of Dee’s vast library, including alchemical books—the contents of which would soon pique the interest of continental practitioners. Kelley used Ripley’s works, including the *Bosome book*, not only as sources of practical information, but as a means of furthering his own relationships with colleagues and patrons: transactions that in turn influenced Ripley’s posthumous continental reception. The resulting circulation of texts allows us to trace, with unusual precision, the spread of English alchemical ideas in the Holy Roman Empire from the late sixteenth century.

## 1 Introduction

For a large part of his life John Dee was engaged in reading about and practising alchemy. His alchemical interests have been most closely studied in relation to his own *Monas hieroglyphica*, in which he set out his alchemical precepts in their most obscure and challenging form. Less has been said, on the other hand, about Dee the reader of alchemical texts (and colleague of other readers), or about Dee the alchemical practitioner (and colleague of other practitioners). This paper focuses on Dee’s relationship with two kinds of alchemist: the authorities whose works he read, and the contemporary practitioners he knew, or may have known. Often the two groups come together, for one interest that Dee shared with many of his acquaintances was an active interest in recovering, reading, and putting into practice the works of past adepts.

The earliest evidence for Dee’s interest in alchemy is his list of the alchemical books he read (but did not necessarily own) in 1556.^1^ This list is largely composed of the classics of Latin medieval alchemy, including works ascribed to Geber, Arnald of Villanova and Ramon Lull, together with a few English works by George Ripley and Thomas Norton. During his travels in Europe during the 1560s, Dee seems to have become acquainted with a wider range of alchemical doctrines, and began to accumulate a substantial library of alchemical literature. These included up to date copies of books by Paracelsus and his followers, and a fine collection of late medieval manuscripts, many retaining Dee’s annotations, as detailed in Robert & Watson’s important reconstruction of *John Dee’s library catalogue* (1990).

The use of this prodigious library by Dee and his contemporaries has been extensively explored by William Sherman (1995, esp. Ch. 2). More specifically, scholars have pondered the extent to which Dee’s alchemical books were read, copied and discussed by others (Bayer, 2005; Keiser, 2007, p. 190; Webster, 1979, p. 323; cf. Sherman, 1995, pp. 44–45). Given the number of duplicate copies of Paracelsian works in his library, Clulee (1998) posits that Dee may have contributed to the dissemination of Paracelsian doctrines in England, perhaps by loaning out copies or using them for teaching.^2^ This suggestion is supported by the names of various individuals, labelled ‘discipulus’, inscribed in some of these books. Such activity would indicate a significant degree of expertise on Dee’s part. Yet, as Clulee notes, it is difficult to gain a clear sense of Dee’s alchemical thinking, since his writings do not in general deal with alchemy.^3^ His most extended treatment of the subject is the *Monas hieroglyphica* (1564), dedicated to the Holy Roman Emperor Maximilian II: a work that reflects primarily medieval and Neoplatonic rather than Paracelsian influences, and offers at best an obscured view of Dee’s practical alchemical interests.^4^

For Dee’s interest was not confined to the written word—his collections at Mortlake included chemical materials and apparatus, and appended to the house were several outbuildings where he and his assistants practised alchemy.^5^ Traces of this activity now survive only in textual form: in manuscript notes of alchemical procedures, practically-oriented marginalia, and a few contemporary recollections.^6^ Like the issue of Dee’s alchemical influence, the question of how Dee’s books related to his practice is one that can only be partially answered, through sifting diffuse and fragmentary sources.

In investigating both questions, I shall focus on a single, little-studied thread from Dee’s seemingly diverse alchemical interests: the late medieval tradition of pseudo-Lullian alchemy, as popularised in works by or attributed to George Ripley (d. c. 1490). In the second half of the sixteenth century, ‘Ripleian’ writings were well known in his native land, but less so in East Central Europe. This strand therefore stands out in relatively high relief against the rich backdrop of continental alchemy, enabling us more easily to pick out clues to its dissemination. The circulation of Ripleian texts coincides in time with the arrival of Dee and his associate Edward Kelley in Bohemia during the 1580s. Indeed, the spread of Ripley’s alchemy in mainland Europe is intimately related to the circulation of Dee’s books and Kelley’s burgeoning alchemical practice. The travels of the English party therefore leave us clues to the relationship between Dee and two alchemists: the living Kelley and long-dead Ripley.

## 2 Dee, Ripley and pseudo-Lullian alchemy

From the mid-fourteenth century, a body of alchemical texts posthumously and pseudonymously attributed to the Majorcan philosopher Ramon Lull (1232–1316) became increasingly influential on the theory and practice of western alchemy. Detailed studies by Michaela Pereira (1989, 1992) have identified over 140 works in the corpus, encompassing a host of alchemical applications, from the transmutation of metals to the production of medicinal elixirs and the manufacture of precious stones. Pseudo-Lullian techniques and doctrines proved popular in England from the fifteenth century onwards, and were employed in a number of attempts by English alchemists to obtain the support of noble and royal patrons, including an application to Henry VI (Pereira, 1998), and, a century later, numerous proposals addressed to Elizabeth I and her Lord Treasurer, Lord Burghley.^7^

As might be expected given the size of the corpus, pseudo-Lullian alchemical operations are many and varied. However, English commentaries on this tradition frequently invoke two powerful waters, sometimes described as ‘fires’, with contrary actions.^8^ One, the ‘fire against nature’ (*ignis contra naturam*) is a powerful and toxic corrosive, valuable for gold-making but deadly if ingested. The second, ‘natural fire’ (*ignis naturae*), embodies the vegetative power of nature, and may be used to heal both metals and the human body. Various works, describe the use of these fires in manufacturing the ‘mineral’ and ‘vegetable’ stones.^9^ While the former employs corrosive mineral acids in its manufacture, the potable vegetable stone is made using products derived from wine: vinegar, tartar, and spirit of wine (Rampling, 2009, 2010).

One basic recipe for the vegetable stone, found in hundreds of permutations in alchemical literature, employs a metallic body called ‘sericon’ as its prime matter: usually taken to denote minium (red lead). The minium is dissolved in strong wine vinegar, and the resulting solution heated until a thick gum remains in the bottom of the glass.^10^ When distilled, the gum yields a white vapour, the *fumus albus*, which is collected in a receiver and condensed to form a liquid, which is then subjected to further procedures. Owing to the circumstances of its manufacture, the vegetable stone was regarded as combining both mineral and vegetable qualities. In this form, it was regarded as a safe and legitimate product for human consumption, and therefore provided one basis for the medicinal *aurum potabile*—an objective which cannot have been assisted, in practice, by the use of toxic lead compounds.

I have elsewhere described this lead-and-vinegar method as ‘sericonian’, to distinguish it from approaches based on different ingredients and processes (Rampling, 2010, pp. 128–129). In England, this approach was commonly associated with the figure of George Ripley, a canon-regular of the Augustinian priory at Bridlington in East Yorkshire who flourished in the 1470s, as attested by the colophons of his best known works, the *Compound of alchemy* (1471) and *Medulla alchimiae* (1476).^11^ In the *Medulla*, for instance, Ripley gave instructions for the manufacture of the vegetable stone from sericon, and its subsequent combination with the mineral ‘fire against nature’ (a corrosive mixture of cinnabar, vitriol and saltpetre) to produce a powerful transmutational elixir—the *aqua composita*, or compound water (Ripley, 1649, pp. 143–145, 170–172).

Doctrines of both Lull (or ‘Raymond’) and Ripley would have been familiar to John Dee from the earliest days of his interest in alchemy, as appears from the list of alchemical books he read in 1556. These include ‘Quinta essentia Raymundi Lulli’, the abbreviated title of the *Liber de secretis naturae seu quinta essentia*, a key work in the pseudo-Lullian corpus, and the first to employ the ‘quintessence’ (highly rectified spirit of wine) for both medicine and transmutation.^12^ Dee owned at least six copies of this work by 1583, together with many other items from the corpus.^13^

The list also includes an unnamed text, labelled ‘Ripley, anglice.’^14^ By 1556, a variety of Ripleian treatises would have been available in English, although none had as yet been printed. Ripley’s famous *Compound* was composed in English verse, while the *Medulla* was translated into English in 1552 as the *Marrow* or *Mary of alchemy*. Several other Latin texts attributed to Ripley, including the *Philorcium alchymistarum* and *Concordantia Guidonis et Raymundi*, were translated around the same time, while two other well known treatises, the *Pupilla alchimiae* and *Accurtations of Raymond*, seem to have been originally composed in Middle English. All circulated widely in the second half of the sixteenth century.^15^

By the far the most popular of Ripley’s works was his Middle English poem, the *Compound*, or ‘Twelve gates.’ Dee later owned a mid-sixteenth-century copy of this work which he annotated on 21 December 1595 (Bodleian Library MS e museo 63, fols. 41r–65r). It is likely that he was familiar with the *Compound* long before this—by the 1560s, it was already one of the best known alchemical works in England. Yet Dee’s interest was not confined to Ripley’s poetical masterpiece: he also copied out several Ripleian prose works in his own hand, all of which may be distinguished by their practical, ‘sericonian’ character. These include the *Accurtations of Raymond*, a theoretical treatise accompanied by a collection of recipes, which was transcribed (at least in part) by Dee, with copious annotations, in Wellcome Library MS 239 (pp. 1–44).^16^ Like the pseudo-Lullian waters it describes, this text is a composite, which accreted over time from originally disparate elements (Rampling, 2009, chap. 4; 2010). However, by the second half of the sixteenth century it was generally regarded as having been compiled by George Ripley, an association that doubtless stemmed from the distinctively ‘Ripleian’ flavour of both the theorica and practica (which include several sericonian recipes), as readers often observed in marginalia. In Dee’s copy, for instance, one note compares a passage on fermentation with the *Compound*: ‘Riplay in his 12 Ga[tes] in the Chap. of Ferm[entation]’ (MS 239, p. 33).

Dee seems also to have drawn on Ripley when translating his textual studies into practical procedures. Bodleian Library MS Rawlinson D.241 is an alchemical notebook, written partly in Dee’s hand, that details experiments carried out between 22 June and 6 October 1581. This account of Dee’s daily practices and observations implies that Dee—no armchair alchemist—was willing to get his hands dirty. At one point, he describes how he obtained over 10 oz of ‘quick mercury’ from a sublimate, ‘by my diligence in pressing the soft stuff || betwene my fingers partly: and by washing it | destilled vineger.’ The result was, ‘a faynt fyne slick or slyme: which I yet | left in an stone Cha*m*ber pot in the vyneger, farder | to try what wold com*m*e of it’ (fol. 3r–v). In extracting this slimy ‘mercury’, Dee was guided by an earlier authority:

Whereby I perceyued that an [ounce] had byn enough | or 6 [drams] rather, to kepe the water in hand | and not to overcharge it, as Riplay in philortiu*m* warnes of. (Ibid., fol. 3r)

The reference is to a passage in the *Philorcium*, in which Ripley advises his disciples to take care when adding liquid to their work:

Draw a water from the same stone using an alembic, and with that water dissolve the stone by alembic, and with that water dissolve the stone infinite. But dissolve it by little and little, and you shall not incontinently suffocate it with water, for if it is correctly regulated you may with one pint of water make, if you wish, an infinite quantity of water.^17^

Ripley was clearly regarded—at least by Dee—as having something more than theoretical application: his recipes and recommendations assigned practical value almost 90 years after his death.

Following the arrival of the scryer Edward Kelley in his household in 1582, Dee’s alchemical pursuits increasingly relied on spiritual as well as temporal authorities. Within two years of making the notes in MS Rawlinson D.241, Dee had relocated himself, his family, and many of his alchemical books to East Central Europe, where he and Kelley attempted to establish themselves first in Cracow and later in Prague, guided by the advice of their angelic interlocutors (Harkness, 1999; Parry, 2011). Yet Dee’s practical interest in alchemy continued. Furthermore, his continental travels coincided with a new phase in the reception of Ripleian alchemy: one that would culminate 40 years after his death with the publication of Ripley’s *Opera omnia chemica* (1649) in Kassel. When compiling the twelve texts that constituted this volume, the editor, Ludwig Combach, was concerned to secure reliable exemplary manuscripts—at least one of which was linked to Dee and Kelley, and their contacts within the Empire. These connections not only shed light on the alchemical activities of Dee and Kelley in Bohemia, but also suggest that these played a role in shaping Ripley’s own posthumous reception.

## 3 Ripley and the ‘Kelley circle’

Dee, Kelley and their families left England on 21 September 1583, in the company of Albrecht Łaski, palatine of Sieradz, whose pretensions to the throne of Poland had received encouragement in the angelic conversations mediated by Kelley at Mortlake. Following an inauspicious start in Cracow and at the imperial capital of Prague, the Englishmen eventually settled in Třeboň (Wittingau) as clients of Vilém of Rožmberk (1535–1592), one of the greatest magnates in Bohemia, and like the Emperor, Rudolf II, a patron of alchemists.^18^ In time, Kelley’s reputation as an alchemist enabled him to obtain imperial favour and a position at Rudolf’s court, where his claims of noble Irish descent were validated by the conferral of a knighthood, and the opportunity to amass considerable property.

Until his death in poorly-documented circumstances, probably on 1 November 1597 (Prinke, 2010), Kelley pursued a spectacular alchemical career in Třeboň and Prague. Indeed, by 1587 his own claims to alchemical expertise had eclipsed those of his erstwhile ‘senior partner’, Dee, securing the favour of both Rožmberk and Rudolf II and attracting interest from Elizabeth I and Lord Burghley in England. The reasons for his success are as elusive as they are incontrovertible. English and Bohemian sources testify to Kelley’s extraordinarily convincing alchemical transmutations, some involving the active participation of the Emperor himself. While the extent to which Kelley himself believed in the authenticity of alchemy remains unclear, it is certain that many of his contemporaries regarded him as a legitimate adept, whose successes were converted into status, wealth, and real estate.

Kelley’s rise is mirrored by an increasing interest in the works of George Ripley in East Central Europe, which seem to have made their Bohemian debut at around the same time. For instance, nine Ripleian works were transcribed in Bohemia in 1605–06 by the alchemist Simon Thadeas Budek, including a Latin translation of the *Compound*.^19^ Budek’s keen interest is attested by dense annotations and cross-referencing, and by the provision of a 31-folio catalogue listing terms and materials employed in Ripley’s works.^20^ The manuscript also includes an intriguing reference to Kelley, reporting that the disgraced Englishman committed suicide following a failed attempt to escape from Rudolf’s custody (ÖNB 11133, fol. 393v; Hausenblasová & Purš, 2009, p. 78).

Such connections were not lost on Ripley’s later editor, Combach, who attributed the Latin translation of both the *Compound* and two other works (the *Epistle to Edward IV* and the *Clavis aureae portae*) to Kelley.^21^ For the first two cases at least, this attribution is spurious. The same translations of the *Compound* and *Epistle* were already circulating in France by the early 1570s, as the *Liber duodecim portarum* (‘The book of the twelve gates’) and the *Epistola ad Regem Eduardum* respectively.^22^ An abbreviated, prose version of the *Liber* was printed in Frankfurt in 1595, edited by the Paracelsian physician Bernard Georges Penot (*c*. 1522–1620), while a fuller version was published in Leiden in 1599 by Penot’s friend, the Protestant pamphleteer Nicholas Barnaud (*c*. 1539–1604?), in his *Quadriga aurifera*.^23^ Combach’s own edition, although the most complete to date, was based on the same early translation.

Combach’s attributions might therefore be dismissed as an attempt to promote Ripley’s authority by linking it to the celebrated Kelley. Yet surviving manuscripts suggest that Kelley’s own fascination with Ripleian alchemy may have helped stimulate wider interest in the medieval English alchemist, subsequently contributing to the *Liber*’s appearance in print. Several of the individuals involved in the later dissemination of Ripley’s works were staying in Prague at roughly the same time as Kelley, and in circumstances conducive to the sharing of alchemical ideas, or, for that matter, texts.

Penot and Barnaud actually met in Prague some time after 1586, while Barnaud was a guest of another alchemical enthusiast, the emperor’s physician, Thaddeus Hájek (1525–1600). During this time they collaborated on a ‘Practica alphabetica’, which—as Penot later reported—Barnaud ‘wanted to be arranged according to the time honoured model.’^24^ By this, Barnaud seems to have had in mind an alphabet of the kind found in the pseudo-Lullian *Testamentum*, in which letters denote ingredients and chemical processes. The alphabet, published in Penot’s *Apologia* (1600, pp. 85–96) as ‘Rubri & Albi vini a terra foliata extractio’ (‘The extraction of red and white wine from foliate earth’), yields a fairly straightforward sericonian recipe, beginning with the dissolution of the calcined ‘green lion’ in vinegar to create a gum.

There would have been plenty of opportunities for both men to meet the English visitors. Barnaud, like Dee and Kelley, became a client of Vilém of Rožmberk, while his host, Hájek, had housed the Englishmen during their first visit to Prague. Barnaud would eventually dedicate his edition of the *Liber* to another Bohemian acquaintance: Sir Edward Dyer (1543–1607), an English courtier and poet sent to Prague on at least three occasions between 1588 and 1590, partly in repeated attempts to persuade Kelley either to return to England or to teach him alchemical secrets.^25^ Kelley’s distinctive presence on the Bohemian alchemical scene is also recorded in Penot’s *De denario medico* (1608), which refers to transmutations carried out by Kelley during Penot’s own sojourn in Prague (ibid., pp. 90, 139).

Since Penot’s and Barnaud’s exemplary manuscripts are no longer extant, we cannot know for certain whether their interest in Ripley’s *Compound* was fanned by the presence of the English visitors in Prague. However, there is more tangible evidence that Kelley took an active role in promoting Ripley’s works amongst well placed colleagues. This includes the appearance of a second, alternative translation of the *Liber*, composed in elegiac verse by the German alchemist Nicolaus Mai, apparently at Kelley’s instigation.

Mai, or Maius, was well connected in both political and alchemical circles.^26^ In 1601, Rudolf II appointed him to the position of *Appellationsrat*, prefect of the silver mines at Joachimsthal—a post previously vacated by another alchemist, Sebald Schwaertzer. From there he corresponded with another alchemical patron, Moritz, Landgraf of Hesse-Kassel. In one letter he apologises for being prevented from visiting Moritz, since the emperor requires his services as an envoy to the Counts of Mansfeld.^27^ In lieu of his person, he reveals an approach to alchemical transmutation that, in characteristic sericonian style, begins with lead and vinegar.^28^

Mai also maintained links with other alchemical practitioners. Penot dedicated a tract in *De denario* to him, addressing him as a councillor to the Elector of Brandenburg (1608, p. 127). Mai contributed a eulogistic poem to the *De signaturis internis rerum* (1609) of the Paracelsian chymist Oswald Croll, and was the addressee of one of the letters collected in the *Rerum chymicarum epistola* (1595) of the Saxon alchemist and physician, Andreas Libavius.^29^ He was also acquainted with both Dee and Kelley. Dee wrote to him on 14 November 1586 (Dee, 1582, fol. 88v), and he visited Třeboň for a few days in 1589, as Dee recorded in his diary: on 26 January 1589, ‘Mr Maius cam[e] to visit vs’, and four days later, ‘Mr Maius went away’ (ibid., fol. 125r). Mai was also connected to the Kelleys: he later composed an epitaph for Kelley’s widow, Joan.^30^ Mai and Kelley seem to have been drawn together by their common interest in alchemy: an interest manifested in Mai’s decision to translate Ripley’s *Compound* into verse for presentation to the Emperor.^31^

In his verse dedication, Mai complains that while his own age is not without learning, it struggles to regain the wisdom of earlier authorities:

One man, since he grasps nothing, condemns the most learned writings of the wise ancients, calling them barbarous writings; another despises chymistry as false, and believes the books of Ripley vain dreams.^32^

The Emperor, as patron and guardian of alchemists, of course knows better. Yet, Mai explains, he actually has two reasons for completing the translation:

The first is that you understand the arts and are favourable to philosophers; you nurture artificers. The other is that Kelley, than whom no one is more excellent, ordered this work to be turned into Latin verse.^33^

The Englishman’s influence is underscored by the inclusion of a distichon attributed to Kelley, inserted between Mai’s dedication and Ripley’s Prologue:

Kellaeus lectori

Cuicquid philosophum congesserat ordine Turba,

patria Rypplaei carmine Lingva dedit:

Haec eadem Maius, calamo facit esse Latino,

Hinc notus Rypplai est, Notior ille magis. (Ibid., fol. 5v)

[Kelley to the Reader

Whatever the crowd of philosophers has gathered in order,

Ripley in his father tongue has given to song:

Maius fashions the same into Latin with his pen,

Hence Ripley is esteemed—but Maius even more.]

This artful verse, with its pun on the shared meaning of *maius* and *magis* (‘greater’), suggests that Kelley was able to trade on Ripley’s reputation as a means of forging or strengthening relationships with other adepts. Besides encouraging Mai to compose a more elegant verse translation of the *Liber*, Kelley also compiled a number of Ripleian works for presentation to a Silesian patron. These survive in a Latin manuscript, Książnica Cieszyńska SZ DD.vii.33: a compendium of Ripleian treatises transcribed in Prague between May and July 1592 by one ‘Jan Kapr.’^34^

Jan Kapr of Kaprštejn, also known as John Carpe or Johannes Carpio, was an administrator responsible for Rudolf’s vineyards.^35^ As Carpio, he is mentioned several times in Dee’s diaries as one of Edward Kelley’s known associates: a regular visitor to Třeboň, who may have assisted Kelley in his laboratory (Dee, 1998, p. 230). His latinized name appears in several alchemical manuscripts now in the Royal Library of Copenhagen, written in English hands and connected with Kelley (Bäcklund, 2006). Bäcklund argues that these manuscripts ‘strongly suggest … the production of English alchemical manuscripts stemming from a circle around Dee and Kelley in Prague’, a circle that was active from 1588–89, and may have continued even after Kelley’s death until the death of Rudolf II in 1612 (ibid., p. 306). The existence of such a circle is further supported by the transcription of SZ DD.vii.33, which suggests that in addition to practical assistance, Kapr had access to Kelley’s alchemical books, and may have served him as an amanuensis. At the conclusion of Ripley’s *Medulla*, he has added a note, indicating that the collection was compiled at Kelley’s behest:

Edward Kelley wrote this book out of kindness and love for his most sincere friend, the noble lord Karl von Biberstein, 2 August in the year 1589: whom he wishes to have known as his adopted philosophical son, and to be esteemed above all other mortals.^36^

The recipient of this gift, the nobleman Karl von Biberstein, or Karl of Biberštejn (1528–1593), was a Silesian official and imperial councillor, who twice served as master of the Bohemian mint.^37^ Like Mai, Biberstein was therefore well positioned both socially and economically, and given his position might have been expected to have a particular interest in novel metallurgical processes, including alchemy. As Dee recorded in the diary, Biberstein visited Třeboň on 26 March 1587, and ‘sent for me to his ynn to make acquayntanse with’, returning to Třeboň on 16 September (Dee, 1582, fols. 96r, 100v). Although Dee was silent regarding the substance of their conversations, it was apparently Kelley who made the most lasting impression on the mint master: a relationship that was clearly in good health in August 1589, some months after Dee’s own departure from Bohemia.

In adopting Biberstein as his ‘philosophical son’, Kelley assumed the role of his master and tutor, a relationship embodied in the transfer of alchemical knowledge. It is significant that the knowledge Kelley bestowed on his pupil centred on the work of his English predecessor, Ripley. SZ DD.vii.33 includes seven Ripleian works: the original Latin translation of the *Liber duodecim portarum* (fols. 28r–31r, 165v–204r), together with the *Epistola ad Regem Eduardum* (fols. 31v–37r), *Liber de mercurio et lapide philosophorum* (fols. 37v–44v, dated 17 July 1592), *Clavis aureae portae* (fols. 45r–62r), *Medulla alchimiae* (fols. 99r–119r), *Pupilla alchimiae* (fols. 139v–146v, dated 14 May 1592), and *Philorcium alchimistarum* (fols. 147r–165r). These are supplemented by other texts, including a treatise attributed to Ripley’s major authority, Raymond, and the Latin text of the *Work of Dunstan*, a theoretical and practical treatise pseudonymously ascribed to the tenth-century archbishop of Canterbury, but actually adapted from another work in the Ripley Corpus—the *Accurtations of Raymond*.^38^

Besides its value as a storehouse of authoritative texts, the compendium thus drew attention to English expertise in the theory and practice of alchemy: a tradition to which Kelley was himself an heir. Ripley’s authority thus underwrote Kelley’s, while his assembled corpus offered a store of intellectual capital that could be disbursed as gifts and deployed as evidence of experience within a long established practical tradition. Yet the benefits of this diachronic relationship were not one-sided. Kelley’s interest in Ripley would have a lasting effect on the European reception of the medieval adept, as may be seen from the subsequent history of the Ripley corpus.

By 1649, Rudolf II and Moritz of Hesse-Kassel were long dead and their alchemical circles dispersed. The Thirty Years’ War had raged through Europe, and a presentation volume of Mai’s *Liber duodecim portarum*, either the Emperor’s copy or Rožmberk’s, was borne away from Prague as booty by Swedish troops.^39^ Another copy, now 4^o^ MS chem. 68, came to rest in the archives of the princely court of Hesse-Kassel, there to be noticed by Ludwig Combach, physician to the former landgrave, Moritz, and his successor Wilhelm. In the preface to the *Opera*, Combach expressed his regret at being unable to publish Mai’s version of Ripley’s poem, since the manuscript was not his own.^40^ Fortunately, he still had the earlier translation to fall back on, which he believed to have been translated by Kelley, found in a manuscript previously owned by Mai.^41^

This manuscript, now 4° MS chem. 67, is a compendium of Ripleian texts, probably compiled in Prague some time after 1600, densely annotated and amended by several scribes. One of the annotating hands is that of Mai, while the compendium also includes one of Mai’s Latin verses, ‘Ænigma M. Nicolaii Maii’ (fol. 183r). Although Mai’s is not the principal hand, we would have to guess that the scribe, too, had some relationship with Kelley. The collection includes a recipe heard from Kelley’s own lips (‘Ex ore EK’); the ‘Deposition of Edward Kelley, Englishman’, dated Galway, 10 March 1593 (testifying to Kelley’s noble Irish lineage); and several extracts apparently taken from letters (one dated Prague, 20 June 1587).^42^

To Combach, these Kelleian connections may have been at least as valuable as the texts they adorned, to judge by the fact that he published the epistolary fragments two years earlier than the *Opera*, in his *Tractatus aliquot* (Combach, 1647, pp. 31–33). Their provenance was assured since they came from one of Councillor Mai’s own manuscripts, as Combach asserted in the preface to the *Tractatus*.^43^ Later, he returned to the manuscript again when preparing the *Opera*. It provided the primary exemplar for his edition of the *Liber*, and is the second of two manuscripts used in preparing the *Medulla*.^44^ In total, MS chem. 67 includes eight of the twelve texts included in the *Opera*.^45^ All eight of these recur in Budek’s Bohemian compilation, while six were also included in Kelley’s gift to Karl von Biberstein.

Combach may therefore have felt that he had good grounds for adding Kelley’s name to Ripley’s works. The references to ‘E.K.’, coupled with a complete copy of the *Liber* in a manuscript previously owned by Kelley’s known associate, Mai, could well have persuaded Combach that one English adept, Kelley, took a hand in the dissemination of another: Ripley. In preparing the *Opera*, Combach thus built on the activities of a network of practitioners based in Bohemia some 60 years earlier—a circle linked to Kelley, and characterised by its interest in the English Canon of Bridlington.

## 4 Ripley in practice

Within the circle of Kelley’s alchemical clients and correspondents, John Dee himself has so far played a surprisingly modest role. Although his *Monas* was discussed and imitated in print and manuscript (including a reference to ‘Liber Monadis J. Δ’ in MS chem. 67, fol. 1v), Dee’s name appears less frequently than that of Kelley in relation to alchemical receipts.^46^ The disparity may be partly explained by Kelley’s celebrity as a successful adept—a reputation which naturally placed a premium on recipes associated with his name—and partly by Kelley’s own strategy of actively disseminating alchemical texts, ranging from authoritative treatises to his own poems, letters and receipts.^47^

What, however, was the source of Kelley’s bibliographical knowledge? Little is known of his reading habits prior to his departure from England. On one famous occasion he brought Dee a book: the mysterious ‘Book of Dunstan’ that he claimed to have discovered, together with a sample of powder, following angelic guidance.^48^ In general, however, the evidence suggests that he owed such knowledge to Dee, who lent him books, and translated the *Opuscule* of Daniel Zeccaire for him out of French (Dee, 1582, fol. 105r). Given the scale of Dee’s alchemical interests, manifested both in his books and his laboratories, it seems probable that some of the information Kelley shared with his ‘circle’ also originated from Dee’s famous library. When Dee set out for Europe, he was accompanied by approximately 800 of his books, some of which are mentioned in his diaries.^49^ Although we do not know for certain whether these included any of Ripley’s works, we can infer that they did, and that these works were shared within a network of which only vestigial traces now remain. This conjecture is supported by the case of a lesser known Ripleian work, the *Viaticum*.

The *Viaticum seu varia practica Georgii Riplaei* (‘Viaticum, or various practices of George Ripley’) is one of the works included in Ripley’s *Opera* (1649, pp. 337–65). Unlike the widely travelled *Liber duodecim portarum*, it is possible to set a *terminus post quem* for this work’s appearance in East Central Europe, for the *Viaticum* includes several processes extracted from a manuscript that was only discovered in 1574, by the English alchemist Samuel Norton (1548–1621).

On 20 July 1577, Norton, son of the Somerset gentleman Sir George Norton, dedicated his alchemical treatise, the *Key of alchemie*, to Elizabeth I. Here he described his discovery of an old Latin commonplace book, containing Ripley’s personal notes and jottings—his daily ‘Bosome book’:

Although it fortuned mee in manner vnloked for, to hitt vpon the secret bosome booke of Riple, wherby the true grounds are discovered, Of w*hi*ch havinge by profe found so many to be true, and little doubtinge of the accomplishment of the rest; I thought it but a point of dutie to reveall and vppen the Secrets heereof vnto y*ou*r Highnes. (Getty Research Institute MS 18, Vol. 10, Pt 2, p. 7)

Norton says nothing of the provenance of this manuscript, which is apparently no longer extant. The Latin text of the original survives in only a single, later copy, now British Library MS Harley 2411. With the intention of presenting its contents to the Queen, however, Norton set about translating the collection of treatises, poems and practical recipes into English.^50^ This plan was subsequently amended, as Norton decided instead to incorporate practical information from the *Bosome book* into his own treatise, the *Key*.

Several copies of Norton’s English translation, dated February 1573 (i.e. 1574),^51^ survive, together with various ‘hybrid’ recipes excerpted and adapted from this translation. Dee was certainly familiar with some of these English redactions: Bodleian Library MS Ashmole 1486 (Part V) contains his own transcriptions of two of them, the *Whole work of the stone philosophical* (pp. 1–18, here titled ‘George Ryppleys bosome booke or Vade mecum’) and the *Practise by experience of the stone* (pp. 19–25).^52^ Dee’s annotations suggest that he was attempting to make practical sense of the recipes (on p. 1 he interprets ‘sericon’ as antimony), which he had grouped beneath the well known aphorism, ‘Liber librum apperit’ (‘The book opens the book’).

While Norton’s English translation seems to have circulated only in England, extracts from the original Latin *Book* were soon available in continental Europe as components of the *Viaticum*: a collection of short, practical tracts gleaned from various sources and gathered under sixteen subheadings. Of these, at least eight are items apparently extracted directly from the *Book*, as appears from comparison with the most complete surviving copy of the latter, MS Harley 2411. The *Viaticum* also shows evidence of revision, most obviously in ‘Oleum verò Solis fiet’ (‘Oil of the sun [i.e. gold] will truly be made’). Although framed as a single, practical process, this short text is actually a reworking of 31 of the *Notable rules from Guido*: a collection of 45 aphorisms supposedly gathered by Ripley from the works of Guido de Montanor, and a component of the *Bosome book*.^53^ Two further sub-sections of the *Viaticum*, ‘Elixir vitae’ and ‘Virtutes huius quintae essentiae hoc modo poterunt probari’ (‘The virtues of this quintessence may be proved in this way’) have been adapted from another Ripleian work, the *Elixir vitae*, that circulated widely in English in the later sixteenth century.^54^ Yet another, ‘Sequitur aliud opus’, is extracted from the *Accurtations of Raymond*. The *Viaticum* is therefore a compendium, prepared by a compiler who was clearly familiar with a variety of Ripleian works, including the original version of the recently discovered *Bosome book*.

Although a few later manuscripts of the *Viaticum* survive in English archives, the distribution of surviving copies suggests that the work may actually have been compiled in Bohemia. The earliest manuscript copies date from around the turn of the century, in the compilations of Nicolaus Mai (MS chem. 67) and Simon Budek (ÖNB Codex 11133). The presence of these excerpts in Prague within a few decades of Norton’s discovery merits some investigation, since their appearance of course postdates the arrival of Dee and Kelley in Třeboň.

A clue is provided by the copy in MS Harley 2411. This early seventeenth-century manuscript includes a recipe entitled ‘Magna philosophorum corrosiva’ (‘The great corrosive of the philosophers’). This begins with the distillation of vitriol with ‘salt of sericon’, resulting in a curious residue:

When our menstrual ‘mercury’ ascends from the sericon by the violence of the fire, a certain part of it is found cleaving to the side of the flask after the complete distillation and cooling of the glass, like salt and of crystalline appearance. And that crystalline earth is a fixed material, and apt to receive any form whatsoever. Let it therefore be gathered and kept. And the form of this earth is like mercury sublimed, and therefore shines brightly … This secret I learned through practice: G[eorge] R[ipley], as God is my witness.^55^

Beneath, the scribe has sketched a small picture of a flask and receiver, depicting the crystalline residue as a ring around the inner circumference of the flask. A note adds, ‘So in a circle aboue the matter was the cleare matter lyke [mercury]’ (MS Harley 2411, fol. 55r). The picture is dated 1588, while additional information is supplied by a marginal note:

I saw the same on 8 February 1588 (new style) in Trebon in Bohemia. From 2 lb. of sericon dissolved in distilled vinegar, and by means of spirit of wine cleansed of much sediment, came 4 oz. of red wine or oil. J:D. E:K.^56^

These notes apparently describe a sericonian experiment carried out by Dee and Kelley in Třeboň, together with its results: the red oil and crystalline residue, complete with sketch. Since MS Harley 2411 dates from after 1588, it is likely that the scribe copied both recipes and annotations from a copy of the *Bosome book* in the possession of Dee or Kelley.^57^ This conclusion is supported by Dee’s own diary entry for 8 February 1588, which records how,

Mr. E.K. at 9 of the clock after none sent for me to his laboratory over the gate: to se[e] how hee | distilled sericon, according as in tyme past & of late he hard [sic] of me out of Riplay. (Dee, 1582, fol. 111r)

Evidently Dee, like Samuel Norton, did not regard the *Book* merely as an antiquity, but as a practical resource. Indeed, this episode provides a rare glimpse of Dee and Kelley’s alchemical activity in Třeboň, in which the Ripleian recipe, taken from Dee’s copy of the *Book*, was tested by Kelley, and the results recorded in the margins of the *Book* itself. At a later point, both the *Book* and its dated annotations were copied by the scribe of MS Harley 2411.

Kelley’s personal interest in the *Book* seems not to have been confined to replication of its practices. One of the epistolary fragments in MS chem. 67 describes,

A certain golden and silvery hermaphoditic water, which, if you will extract it naturally from a perfect body and an imperfect metal, will give you the water of life, the stinking water and the green lion, in which are all colours, ending in two, white and red. It does not matter what earth your substance comes from (as Guido asserts), as long as it is fixed.^58^

This short passage draws on two axioms of Guido de Montanor.^59^ The first defines three ‘species’ observed in the work: the white fume (*fumus albus*), the stinking water (*aza foetida*), and the green lion (*leo viridis*). However, Kelley has modified his source by substituting ‘water of life’ for the ‘white fume.’ The second axiom, concerning the earth to be used, is another saying of Guido, in this case an exact quote.^60^ Although I have been unable to locate these axioms in any of Guido’s surviving works, both are found in two Ripleian texts: the *Accurtations of Raymond* and the *Notable rules from Guido*. Kelley’s usage is slightly closer to the latter, suggesting that the model for his alchemical composition, as for his laboratory practice, was a component of Ripley’s *Bosome book*.

If Dee had a copy of the *Book* with him in Třeboň, this must be considered a likely source for the extracts that began to appear in Bohemian manuscripts soon after, in the form of the *Viaticum*: a distillation of noteworthy processes from an authoritative source, in which we can discern echoes of Dee and Kelley’s own alchemical practice. This new compilation went on to acquire an afterlife of its own in manuscript and print. The reworked process ‘Oleum verò Solis fiet’, based on Guido’s axioms, may also owe its existence to Dee or Kelley, since MS Harley 2411 includes a copy towards the end of the *Book*, with the note ‘Revised’ (fols. 75r–77r, at fol. 77r). Grouped with other items taken from the *Book* and elsewhere in the Ripley Corpus, this process yielded the *Viaticum*: a ‘new’ Ripleian work with a predominantly continental circulation and an unambiguously practical flavour.

That Dee owned a copy of Ripley’s lost book makes sense when we consider his own network of alchemical acquaintances in England, several of whom were involved in the *Book*’s dissemination. These included Edward Cradock, the Lady Margaret Professor of Divinity at the University of Oxford, who translated one item from the *Book*, the *Somnium*, in June 1582, a year before Dee’s departure abroad.^61^ A new English translation of the *Book* was later commissioned by another friend of Dee, Gawin Smith, a prominent engineer in the service of both Elizabeth I and James I.^62^ This translation, made twenty years after Norton’s, was completed on 24 July 1593 by one Roger Howes, ‘for Mr Gawyn Smithe gentleman.’^63^ Smith, whom Howes styles ‘gentleman Master of her ma*ies*ties Engines’, had visited Dee for several days in Bremen in October 1589, and petitioned the queen on his behalf in July 1590 (Dee, 1582, fols. 134r, 147r).

Irrespective of whether or not Norton’s discovery was an authentic manuscript of George Ripley, these connections suggest that the newly discovered works of England’s master alchemist provoked keen interest among Elizabethan and Rudolfine *cognoscenti*. It is unlikely that Dee, the well-connected bibliophile and alchemical enthusiast, would have remained in ignorance of such a find. Rather, the evidence suggests that he acquired a copy himself, and that he considered it sufficiently valuable to include among the 800 books (a fraction of his vast library) that accompanied him to Bohemia.

Like the original *Book*, Dee’s copy has not survived, although its vestiges remain: in the diary record of Kelley’s practical experiments; in the copy in MS Harley 2411; in Kelley’s letter; and in Bohemian copies of the *Viaticum*. Such traces also hint at a vanished network of readers and practitioners, who circulated and transcribed new and authoritative works, translated them into or out of Latin, and even prepared them for presentation to princely patrons. One outcome of such correspondences was the publication of the otherwise obscure *Viaticum*: a work linked to a great English authority, translated by Norton for presentation to an English queen, harboured by Dee, and tested and revised by Edward Kelley.

## 5 Conclusion

In March 1589, disheartened by his lack of fortune and under pressure by Rožmberk to leave Třeboň, Dee departed from Bohemia with his family, leaving Kelley in possession of the field. Over the previous few years, Dee’s preoccupation with the angelic conversations and their millenarian implications seems to have come increasingly into conflict with Kelley’s practical interest in transmutation—an interest more congenial to his patrons, Rožmberk and Rudolf II, than the specious pledges of the showstone. From 1587, Kelley’s attempts to extricate himself from his role as Dee’s scryer were paralleled by his own increasing success as an alchemist (Dee, 1998, p. 210; Parry, 2011, chap. 17).

Although Dee was unable to muster the same level of support for his own projects, he continued to make some contributions to his colleague’s practice. In an entry dated 28–29 October 1587, a few days after Kelley’s return from a visit to Prague, he noted that ‘Jo. Carp. did begyn to make furnaces over the gate &c.: & he vsed of | my rownd bricks’ (Dee, 1582, fol. 102v). Kapr was apparently engaged in equipping Kelley’s laboratory: we may recall Dee’s earlier reference to Kelley’s experiment conducted in ‘his laboratory over the gate.’ A month earlier, on 28 September, Kelley had visited Dee to ask that they split their supply of ‘[mercury] Animall’, bringing weights with him for the purpose (ibid., fol. 101r). The ‘Diary’ leaves terse records of other alchemical activities, but by this stage Kelley was the dominant practitioner, while Dee, in a reversal of their earlier fortunes, increasingly performed the role of his assistant.

This reverse finds a parallel in Kelley’s deft use of another English alchemist, Ripley, whose works offered a form of intellectual capital to be deployed in Kelley’s own writings, correspondence and laboratory practice. Yet although works like the *Bosome book* informed his alchemical activities, the benefits of the association were not one-sided. The connection with a successful and charismatic practitioner, Kelley, attracted new interest in an author upon whom Kelley himself relied, smoothing the passage of Ripley’s English works through the courts and presses of the Empire. In this trans-generational conference of philosophers, one authority supported another—yet Combach eventually published Kelley’s letters before Ripley’s texts.

Even after Dee’s return to England, the three Englishmen would remain inseparably bound in print. Ralph Rabbards, magistrate and frustrated engineer, published Ripley’s *Compound* in 1591: the first time that an English vernacular alchemical work had been printed in its original language.^64^ In his dedication to Elizabeth I, Rabbards hailed the achievements of English adepts, ‘& especially M. Doctor *Dee* in his *Monas Hyerogliphica*.’^65^ His praise was reserved for these adepts’ ‘depth of learning *Theoricall*’, rather than practical: although he hints at the results that might be obtained if the work ‘were yet executed by any experienced practitioner’ (Ripley, 1591, sig. [A4]v).

While Rabbards doubtless had his own skills in mind, another such practitioner was surely Edward Kelley, whose absent presence is recorded by the inclusion of a poem by ‘Sr. E. K. concerning the Philosophers Stone, written to his especiall good friend, G.S. Gent’ (ibid., sig. ^∗^3r–v). This poem was already available in manuscript: in Copenhagen, Royal Library GKS 242 (fol. 37v), one of a group of documents connected with the Kelley circle in Prague (Bäcklund, 2006). This copy is titled ‘The praise of vniti for frendships sake made by astranger to furder his frende his Conceyts. 1589’, and signed ‘S*i*r Edward Kelle’ (ibid., pp. 300–301). Rabbards’ change of title to include ‘G.S. Gent.’ may perhaps suggest a link with Dee’s friend, ‘Mr Gawyn Smithe gentleman’: the royal projector and reader of the *Bosome book*.^66^ If Smith was indeed the recipient of Kelley’s poem, then we might speculate that he and Rabbards, as fellow engineers with a taste for alchemy, also shared an acquaintance—a possible indicator of the route by which Kelley’s 1589 poem reached the English press only two years later.

The host of coincidences surrounding the publication of Ripley’s works draws attention to the vigorous, scribal transmission of early modern alchemical texts.^67^ The first editions of the *Compound*—by Rabbards, Penot, Barnaud and Combach—were not set in isolation, but lay enmeshed within webs of communication, authority and patronage: a pan-European network in which John Dee, practising alchemist and one of the great bibliophiles of Renaissance Europe, was an enthusiastic and influential participant. This network now survives only in fragmentary form: in friendly dedications, in marginal notes, and in the appearance of particular works in unexpected places. Such clues guide us to the routes by which Ripley’s poem attained a level of success that Dee, indifferently successful petitioner to a host of European monarchs, might well have envied: written for an English king, printed for an English queen, and translated for a Holy Roman Emperor.

## Footnotes

1 ‘Authores Alchymici quos perlegi anno 1556, a mense Julij’, in Oxford, Corpus Christi College MS 191 (fols. 88v–90r), listed in Roberts & Watson (1990, pp. 191–193).

2 For a concordance of Dee’s Paracelsian books with Karl Sudhoff’s *Bibliographia Paracelsica*, see Roberts & Watson (1990, pp. 198–200). See also Webster (1979, pp. 331–332).

3 Clulee (1998, p. 116). In relation to his published works this may have been deliberate. In the ‘Mathematicall Praeface’, for instance, Dee avoids overt references to alchemy, making no reference to the *Monas*, although he does cite his earlier *Propaedeumata aphoristica* (1558), which in its revised version (1568) included some alchemical material: see Dee (1978). Dee also referred to alchemy in his diaries and private correspondence.

4 On the alchemy of the *Monas*, see Josten (1964), Walton (1976), Clulee (1988, pp. 77–115) (1998), (2001) and (2005), Håkansson (2001), Forshaw (2005), Szőnyi (2005, pp. 161–174), Cavallaro (2006).

5 See Clulee (1988, p. 178), and his article in this volume.

6 On Dee’s alchemical marginalia, see Sherman (1995, pp. 89–90), Norrgén (2005). On his relationship with other alchemists see Clulee (2005, pp. 200–201), Parry (2011).

7 On alchemical patronage suits to Elizabeth and Burghley, see: Webster (1979), Feingold (1984), Campbell (2009), Parry (2011).

8 This doctrine may originate in the pseudo-Lullian Testamentum, where *ignis naturae* is discussed in chap. 29 and *ignis contra naturam* in chap. 34: (ps.) Lull (1999, pp. 100; 114–116). The ‘fires’ are discussed in the *Scala philosophorum* (Manget, 1702, pp. 134–147), Ripley’s *Compound* (Ashmole, 1652, pp. 142–143) and *Medulla* (Ripley, 1649, pp. 135–137), and the Ripleian *De ignibus nostris* (CRC 15, in Rampling, 2010), inter alia.

9 These include the pseudo-Lullian *Epistola accurtationis* and many works based upon it, including Ripley’s *Medulla* (cf. Ripley, 1649, p. 138).

10 The gum is sugar of lead, or, in modern parlance, lead acetate. In some cases ‘sericon’ is interpreted as verdigris, or copper acetate. In texts from the mid-sixteenth century onwards, it is often reinterpreted as antimony (Rampling, 2009, chap. 4).

11 Works attributed to Ripley are described in *The catalogue of the Ripley corpus* (henceforth CRC) in Rampling (2010). Works and individual manuscripts are assigned CRC numbers, to which I shall refer in the present essay. The *Compound* is CRC 9; the *Medulla* is CRC 16.

12 The first two books of the *Liber de secretis naturae* borrow heavily from the *Liber de consideratione de quinta essentiae* of John of Rupescissa (*c.* 1310–*c.* 1364). Although John describes using the quintessence to extract medicinal properties from gold, he does not discuss transmutation: Halleux (1981), DeVun (2009). On the *Liber de secretis naturae*, see Pereira (1986, 2002).

13 In Roberts & Watson (1990), the manuscripts M46, M66, M67 and DM94 and printed books 1401, 1404 and 1405 include complete or partial copies of the *Liber de secretis naturae*. Dee’s 1556 list contains two other works attributed to Lull, and several florilegia heavily influenced by pseudo-Lullian doctrines: the *Correctio fatuorum*, *Clangor buccinae*, and *Scala philosophorum*. Other pseudo-Lullian alchemical books in Dee’s 1583 catalogue include items 1401–1407 and numerous manuscripts. Cf. ibid., pp. 8–9.

14 Roberts & Watson (1990, p. 192), suggest that this corresponds to the ‘Ordinale secretum’ (attributed to Ripley in a later hand) in DM10, i.e. Cambridge, Trinity College MS R.14.38. However, this is actually Thomas Norton’s *Ordinal of alchemy*, which might better correspond to the item ‘Norton anglice’ on Dee’s 1556 list.

15 Extant manuscripts are listed in the CRC. The *Philorcium* is CRC 25, *Concordantia* CRC 10, *Pupilla* CRC 27, and *Accurtations* CRC 1.

16 This corresponds to DM90 in Roberts & Watson (1990); CRC 1.9 in Rampling (2010).

17 ‘Trahe aquam de eodem lapide per alembicum, & cu*m* illa aqua solve lapidem per alembicum, & cum illa aqua solve lapidem infinitum, sed solve per modicum & aqua non suffoces incontinenti, quia si rectè regatur cum aquae pinta una facies, si volueris, aquae quantitatem infinitam.’ Ripley (1649, p. 200). Translations are my own unless otherwise indicated.

18 The classic study of Rudolf II’s alchemical interests is Evans (1997, esp. chap. 6). On Dee and Kelley’s activities in East Central Europe, see ibid. (pp. 218–228), Clulee (1988), Harkness (1999), Wilding (2007), Telle (2010), Prinke (2010), Karpenko & Purš (2011), Parry (2011).

19 Österreichische Nationalbibliothek Codex 11133, fols. 361v–67r. On Budek, see Hausenblasová & Purš (2009).

20 ‘Index rerum et verborum in o*mn*ia op*er*a venerabilis Canonici Domini Georgij Riplei Angli.’ ÖNB Codex 11133, fols. 159r–190r.

21 ‘Finis libri 12. portarum, quem cum Epistola ad Regem Eduardum ex rhythmis Anglicis Latinè vertit Eduardus Kellaeus’, Ripley (1649, p. 100); ‘A plerisq*ue* illum, sub titulo Clavis aureae portae, Riplaeo tribui video, atq*ue* hoc nomine ex Anglico idiomate illum vertit Eduardus Kellaeus’, ibid., p. 225.

22 See, for instance, CRC 9.42 and 9.46–47 in Rampling (2010).

23 Ripley (1595, 1599). On Penot and Barnaud, see Olivier (1992–1996), Kahn (2005, 2007, pp. 108–111, 340).

24 ‘Ne huius artis indagatores mirentur, ita nos sub verborum inuolucris artem tradidisse: et enim cum Pragae apud Do: Hegetium Thadeum ageremus praxim alphabeticam, auspicatisumus cum Do: Nicolao Bernado, quam ipse more veterum descriptam ita esse voluit’, Penot (1600, p. 96). See also Olivier (1992–1996, pp. 586–587).

25 Dyer was knighted in 1596. See Sargent (1968).

26 On Mai, see Evans (1997, p. 209–210; 216), Olivier (1992–1996, pp. 609–610), Telle (1991, pp. 176–177), Croll (1998, p. 13). I am also obliged to Oliver Humberg for details of several manuscripts connected with Mai.

27 ‘Venissem cum ablegatis ipse, nisi me Caesaris iussa ad Comites Mansfeldenses legatum, in negotio quodam arduo, moram non ferente, avocarent.’ Letter from Mai to Moritz, 22 January 1603, Jáchymov, in Kassel LB, 2o MS chem. 19 (Pt. 1), fols. 273r–278v, at fol. 273r.

28 ‘Ex quibus collectis sequitur, subiectum naturâ suâ ad dissolutionem Plumbi idoneam aquam acetosam quandam vegetabilem esse oportere.’ Ibid., fol. 275r. On Moritz’s alchemical interests, see Moran (1991).

29 Croll (1609, p. 76), Libavius (1595, pp. 265–272).

30 Bassnett (2006, p. 290). Kelley’s stepdaughter, the poetess Elizabeth Jane Weston (‘Westonia’), in turn addressed a poem to Mai, expressing gratitude for his support: Weston (1606), Vol. I, poem 18.

31 This translation, ‘Georgii Riplaei, canonici angli, XII. Portarum liber, elegiaco carmine editus a Nicolao Maio,’ survives in two manuscripts. The first, Biblioteca Apostolica Vaticana MS Reg. Lat. 1381, is described in Beda Dudík, *Iter Romanum. Im Auftrage des Hohen Maehrischen Landesausschusses in den Jahren 1852 und 1853*, parts i–ii (Vienna, 1855, p. 228). The second, Kassel Landesbibliothek, 4o MS chem. 68, belonged to the alchemical collection of Moritz, Landgraf of Hesse-Kassel.

32 ‘Hic, veterum damnans doctissima scripta Sophorum, | Cum nihil adsequitur, barbara scripta vocat; | Ille, velut falsam Chymiae despicit Artem, | Riplaeiq*ue* libros somnia vana putat.’ 4o MS chem. 68, fol. 4v.

33 ‘Causa mihi duplex: prior est; intelligis | Artes, Philosophisq*ue* faves, Artificesq*ue* foves. | Altera; Kellaeus, quo non praestantior alter, | Hoc Latiis numeris vertere iussit opus.’ Ibid., fols. 4v–5r.

34 ‘Descripta per Johannem Carpionem | Pragae die 14 Maij Annor*um* 1592.’ Cieszyn, Książnica Cieszyńska SZ DD.vii.33, fol. 146v.

35 Kapr’s office was that of ‘perkmistr hor viničných’ or ‘Bergmeister der Weingarten’: Kühlmann & Telle (1998, pp. 165–166, 168), Prinke (2011) and private correspondence.

36 ‘Eduard*u*s Keleus Hunc lib|rum in graciam et amorem | Magnifici domini Caroli de Bi|beistaynn Amici sui integ*er*rimi | fecit. Annor[*um*?] 1589 Augu|sti 2o. | Ipsumq*ue* filium suum philoso|phicum adoptat Praeferendum | Etiam semper omnib*u*s mortali|bus merito suo existimat.’ SZ DD.vii.33, fol. 119v.

37 Biberstein was *Landeshauptman* to the Duchy of Głogów. I am grateful to Rafał Prinke for consulting several Polish records on my behalf.

38 The *Work of Dunstan* is based on a Latin translation of the *Accurtations*, with the references to Ripley’s chief authorities, Raymond and Guido, removed—presumably in order to strengthen the case for authorship by the tenth-century Archbishop, Dunstan (Rampling, 2009, chap. 4). To complicate this picture further, the *Clavis aureae portae* is itself a ‘doctored’ version of the *Work of Dunstan*, to which some further material, including a firm attribution to Ripley, has been added (Rampling, 2010, p. 149). Given Kelley’s relationship to both the *Work* and the *Clavis*, it is conceivable that he may have helped compose either or both of these forgeries. One section of the *Clavis* which duplicates material from the *Work* is not present in SZ DD.vii.33, which also includes the full version of the *Work*. On Dee and Kelley’s interest in Dunstan, see Kassell (2000).

39 Dudík (1855, p. 228). Dudík suggests that the manuscript may have been part of the Rožmberk collection in Prague (see also Evans, 1997, p. 210, n.1). It eventually reached Rome via the royal library of Queen Christina of Sweden, which included alchemical manuscripts from the collections of Rudolf II and Rožmberk. On the fate of Christina’s alchemical books, see Blok (1974); on the diffusion of books and manuscripts from Rudolfine Prague, see Mout (1988), Balsem (2007).

40 ‘Habeo etiam in manibus Riplaei huius duodecim portaru*m* librum Augustissimi Imperat. Rudolfi Secundi jussu à Nicolao Majo carmine elegiaco purissimo redditum, & potuissem adiungere pro meliori captu & intellectu hujus tractatus, sed quia exemplar illud meum non est, non debui absq*ue* vernia abuti concredito mihi beneficio.’ Combach, Preface to Ripley (1649), fol. 7r (dated 10 September 1649). As later translated by Ashmole: ‘[Combach] could willingly have added it to that he *published*, (which was *translated* out of *English* into *Latine verse* by Sir Edw: Kelley) for the better understanding thereof, but that the *Copy* was none of his owne.’ Ashmole (1652, p. 456).

41 See n. 31 above.

42 Ibid.: ‘Ex ore EK. | R*ecipe* [mercur]ium [Jov]is et pone in crucibulum super tripodum’, (fol. 141r), ‘ex epistola K: 20 Junii a*nn*o [15]87 Prahae data’ (fol. 181v), ‘Datum Galuiae | vrbis huius prouinceae principalis .X. die | Martii, anno ab incarnatione Dominj | M.D.XCIII’ (‘Testimonium Eduardi Kellaei Angli’, fol. 143v). The latter text is reproduced in Karpenko & Purš (2011, pp. 532–533). See also Rampling (2011).

43 ‘Insequentes tractatus parvuli … cu*m* fragme*n*tis Kellaei … ex codice ms. Domini Nicolai Maij, Augustiss. quondam Imperatoris Rudolfi II. &c. Consiliarij, excerpti sunt.’ Combach (1647, p. 11).

44 The other is 4° MS chem. 66. See Rampling (2009, chap. 7).

45 The *Liber 12 portarum*, *Medulla philosophiae chemicae*, *Clavis aurae portae*, *Pupilla Alchemiae*, *Terra terrae philosophicae*, *Viaticum seu varia practica*, *Cantilena*, and *Epistola ad Regum Eduardum*. Of the remaining four texts printed by Combach, three (*Liber de Mercurio & Lapide philosophorum*, *Philorcium Alchymistarum*, and *Accurtationes & practicae Raymundinae*) are found in Combach’s second major exemplar, 4o MS chem. 66. I have not identified an exemplar for the remaining item, the *Concordantia* (although this work is mentioned by title in 4o MS chem. 67, fol. 133v). It is also likely that Combach had access to additional exemplars for at least some of the texts named above.

46 One manuscript recipe, ‘Collecta ex ore Johan: Dee & Edoardi Kyllaei Angloru*m*’, is unusual in pairing the two: Leiden Universiteitsbibliothek MS Vossianus chym. Q1, fol. 24r. On the reception of the *Monas* in print, see Clulee (1998), Forshaw (2005).

47 On alchemical texts and manuscripts associated with Kelley, see Bäcklund (2006), Karpenko & Purš (2011).

48 Dee (1998, p. 53), Kassell (2000, pp. 144–147).

49 Roberts (2004). Roberts & Watson (1990) suggest that books marked ‘T’ in Dee’s library catalogue were those he intended to take with him to the continent.

50 ‘[W]*hi*ch bookes I soe provided, that it might Come to your Ma*ie*sties hands, not so much ffor the book it selfe, as for my owne severall practise; w*hi*ch should have beene there vnto annexed, with a right Censure & Judgement of proceedinge in the rest.’ Getty MS 18, Vol. 10, Pt 2, p. 8. On Norton, see Mandelbrote (2004).

51 ‘The Copye of a old Booke, w*hi*ch is thowght to be ye hand writtyng of Mr gorge Rippyle Chan*n*on, translated owt of Latten bye samwell norton Esquy*er* the vth of feberary An*n*o domini 1573’, MS Sloane 3667, fol. 124r. This manuscript is the earliest extant copy of Norton’s English translation of the *Bosome book*.

52 The *Practise by experience* is CRC 26; the *Whole work* CRC 35. The latter was published by William Cooper as the *Bosom-book of Sir George Ripley* (Ripley, 1683), later translated into French as *Le Manuel de George Ripley* (CRC 35.15–16). Cooper’s edition should not therefore be confused with the original *Bosome book*: it presents a version of one set of recipes from the Book, translated (and probably adapted) by Norton.

53 Ripley (1649, pp. 349–354). The *Notable rules from Guido* is CRC 22.

54 Ibid., pp. 359–61. The *Elixir vitae*, a medicinal work, is CRC 12.

55 ‘Quando [mercurius] n*oste*r menstrualis ascendit a Serico|ne per violentiam ignis, quaeda*m* pars ipsius | adhaerens lateribus vasis quasi salina et | crystallina reperit*ur* post distillatione*m* comple|tam et vasis infrigidatione*m*, et illa terra | crystallina, est materia fixa & apta ad | recipiendam qua*m*cu*m*q*ue* forma*m*. Colligat*ur* ideo | & servet*ur*. Et terrae illius figura est qua|si figura arg*en*ti vivi sublimati, et ita re|splendet . . . Haec secreta in practicando didici. Haec | G:R: teste Deo.’ MS Harley 2411, fol. 55r. A similar recipe appears in the *Whole work*, later printed in Ripley (1683). However, the print version glosses ‘sericon’ as ‘antimony’, a reading that appears in neither the original Latin text of the *Book*, nor Norton’s translation. The original process is closer to Ripley’s recipe for a ‘compound water’ in the *Medulla*, which also begins with the distillation of vitriol (an ingredient of the ‘fire of nature’) together with ‘natural fire’ (made from sericon and vinegar): Ripley (1649, pp. 143–145).

56 ‘Ego idem vidi | A*nn*o 1588 feb: | die 8. novo sti|lo Tr*a*eboniae | in Bohemia | Ex 2 lib Se|riconis dissol|uti in [vinegar] dis|tillato, et p*er* | sp*iritu*m vini pur|ificatu*m* a | multis f*a*ecib*u*s | 4 oz erant | vini rubicu*n*|di sive olei. | J:D. E.K.’ MS Harley 2411, fol. 55r.

57 Another note, ‘J:D. Quae tantu*m*|modo vna est’, appears on fol. 18v.

58 ‘[A]qua quaedam argentea & aurea, hermaphrodita, quam si à corpore perfecto & imperfecto metallico à natura extraxeris, aquam vitae, asam foetidam & viridem habebis Leonem, in quo omnes sunt colores, terminati duo, Albedo & Rubedo. De terra non est curandum, modò (ut Guido affirmat) sit fixa.’ In ‘Ex epistola Eduardi Kellaeri Angli data Trebonae 20. Junii anno 1587’, Combach (1647, p. 31). Combach’s source was evidently the fragment in Kassel LB, MS chem 67, fol. 181v.

59 Guido de Montanor, Ripley’s preferred authority after Raymond, is now known predominantly through Ripley’s own writings. See Rampling (2008).

60 Kelley’s version may be compared to the *Viaticum*: ‘De terra autem non est curandum, de qua sit substantia, dummodò sit fixa’, Ripley (1649, p. 350); and Dee’s copy of the *Accurtations*: ‘And that vnderstode well Guydo . . . De terra non est curandum, de qua sit substantia, dummodo fuerit fixa’, Wellcome Library MS 239, pp. 10–11.

61 ‘Thus endeth ye dreame of Sir George Ripley | Chanon of Bridlington. this was translated owte | of laten verse ye 4th day of June a*nn*o 1582 by Mr doctor Cradocke.’ Philadelphia, University of Pennsylvania Codex 111, fol. 43r. On Cradock, see Feingold (1984, p. 86). Schuler (1995) includes an edition of Cradock’s ‘Treatise touching the philosopher’s stone’ at pp. 3–48. The *Somnium* is CRC 28.

62 British Library MS Sloane 3682, f.1^∗^r. Smith later received letters patent from James I which enabled him to style himself ‘cheife Enginer of England’ (‘The true Coppie of a peticion deliuerid to ye L: mayor & | Aldermen of ye Cittie of London by Gawin Smith’, British Library MS Cotton Titus B.V, fol. 273r). For other manuscripts referring to Smith, see Harkness (2007, p. 286, n. 56).

63 MS Ashmole 766 (Pt. 5), fol. 1r. Howes was an experienced translator of alchemica, having also translated copies of works by Petrus Bonus Ferrarius for Smith in 1590: *Introductio in divinam chemiae artem integra* (completed 16 October) and *Pretiosa margarita novella* (30 November), in MS Sloane 3682. Howes comments that the translation was ‘the work of 60 days or thereabout’ (fol. 285r).

64 Rabbards describes how he, ‘hauing these fortie yeares amongst many other most commendable exercises and inuentions of so warlike Engines, founde out diuers deuises of rare seruice, both for Sea and land’, only to lose the credit ‘ignoraunt persons … [who] vainely arrogated the inuention vnto themselues.’ Rabbards, ‘Epistle dedicatorie’ to Ripley (1591, sig. A3v).

65 The prefatory poems include one by ‘J.D. gent: in praise of the Author, and his Worke’ (Ripley, 1591, sig. ^∗^2r), sometimes attributed to Dee, apparently on the basis of the initials: French (1972), p. 82 n. 2. Given Rabbards’ earlier singling out of the *Monas*, Dee’s authorship is nevertheless a possibility: he sometimes wrote English verses, and his brief *Testamentum* was later included in Ashmole (1652, p. 334).

66 The notion receives some tentative support from the appearance of ‘Smith’ among the deleted names identified by Bäcklund (2006, p. 299), in the margins of another manuscript in the Copenhagen ‘Kelley’ group: GKS 1727 4° (*c*. 1593–1595).

67 Even the *Bosome book* left its mark on Rabbards’ edition. The *Compound* is prefaced by a short poem, ‘The vision of Sir George Ripley, Chanon of Bridlington’ (Ripley, 1591, sig. [^∗^4r]). This is actually Samuel Norton’s English translation of a Latin poem, the *Visio*, excerpted from the *Book* (CRC 32).
